# eIF6 promotes the malignant progression of human hepatocellular carcinoma via the mTOR signaling pathway

**DOI:** 10.1186/s12967-021-02877-4

**Published:** 2021-05-20

**Authors:** Liping Sun, Shuguang Liu, Xiaopai Wang, Xuefeng Zheng, Ya Chen, Hong Shen

**Affiliations:** 1 Department of Pathology, School of Basic Medical Sciences, Southern Medical University, Guangzhou, China; 2 Department of Pathology, Nanfang Hospital, Southern Medical University, Guangzhou, China; 3 Department of Pathology, The Eighth Affiliated Hospital, Sun Yat-Sen University, Shenzhen, China; 4 Department of Pathology, School of Medicine, Guangzhou First Peoples Hospital, South China University of Technology, Guangzhou, China; 5 Department of Anatomy, Neuroscience Laboratory for Cognitive and Developmental Disorders, Medical College of Jinan University, Guangzhou, China

**Keywords:** EIF6, MTOR, Hepatocellular carcinoma, Cell proliferation, Prognosis

## Abstract

**Background:**

Eukaryotic translation initiation factor 6 (eIF6) has a crucial function in the maturation of 60S ribosomal subunits, and it controls the initiation of protein translation. Although emerging studies indicate that eIF6 is aberrantly expressed in various types of cancers, the functions and underlying molecular mechanisms of eIF6 in the pathological progression of hepatocellular carcinoma (HCC) remain unclear. This study aimed to evaluate the potential diagnostic and prognostic value of eIF6 in patients with HCC.

**Methods:**

HCC samples enrolled from The Cancer Genome Atlas (TCGA), Gene Expression Omnibus (GEO) and our cohort were used to explore the role and mechanism of eIF6 in HCC. The diagnostic power of eIF6 was verified by receiver operating characteristic curve (ROC) analysis and its prognostic value was assessed by KaplanMeier analysis, and then related biological functions of eIF6 were determined in vitro and in vivo cancer models. In addition, potential molecular mechanism of eIF6 in HCC was unveiled by the gene set enrichment analysis and western blot assay.

**Results:**

We demonstrated that eIF6 expression was markedly increased in HCC, and elevated eIF6 expression correlated with pathological progression of HCC. Besides, eIF6 served as not only a new diagnostic biomarker but also an independent risk factor for OS in HCC patients. Functional studies indicated that the deletion of eIF6 displayed tumor-suppressor activity in HCC cells. Furthermore, we found that eIF6 could activate the mTOR-related signaling pathway and regulate the expression level of its target genes, such as CCND1, CDK4, CDK6, MYC, CASP3 and CTNNBL1, and these activities promoted proliferation and invasion of HCC cells.

**Conclusions:**

The findings of this study provided a novel basis for understanding the potential role of eIF6 in promoting tumor growth and invasion, and exploited a promising strategy for improving diagnosis and prognosis of HCC.

**Supplementary Information:**

The online version contains supplementary material available at 10.1186/s12967-021-02877-4.

## Background

Hepatocellular carcinoma (HCC) accounts for 8090% of liver cancer cases and ranks as the third leading cause of cancer-related deaths worldwide [1]. Pathologically, HCC frequently derived from mature hepatocytes and is a highly heterogeneous malignancy; there are major risk factors associated with HCC, including HBV and HCV infection, alcohol abuse, and fatty liver disease [2]. Although its survival rate has reduced to less than 20%, which is primarily attributed to enormous progress in early diagnosis, systemic immunotherapy and surgical technics, the mortality rate of HCC remains almost equal to the incidence rate, which indicates a lack of effective early diagnosis and treatment [3]. Consequently, elucidating the potential molecular pathogenesis involved in HCC is pivotal for improving therapeutic effectiveness and prognosis.

In recent decades, as scientists have been concerned with the origin and etiology of tumor cells to discover an effective means of prevention and treatment from the pathogenesis of tumors, dysregulation of protein translation and cancer-related pathways have been confirmed to be involved in development and progression of tumor and could affect the survival of patients diagnosed with cancer [4, 5]. All the time, eukaryotic translation initiation factors (eIFs) are be considered as rate-limiting step for protein translation initiation and their disorders in the expression and location are interpreted as a cause of cancer progression and malignant biological behavior of cycling cells [6, 7]. In recent years, emerging evidence has shown that eIFs play a vital role in tumor pathology. For example, when disordered or overexpressed, eIF4E can trigger neoplastic transformation and result in tumorigenesis via regulating the conventional translation rate of specific proteins, and patients with eIF4E-high expression tend to have a relatively dismal prognosis [8, 9]. In contrast, obvious deletion of eIF1 is revealed in pancreatic cancer samples compared with non-neoplastic pancreatic tissue [10]. From this perspective, interpreting the function of eIFs in protein synthesis can be conducive to discovering the molecular mechanisms involved in cancer progression.

Eukaryotic translation initiation factor 6 (eIF6), a conserved 27kDa protein present in eukaryotes, was discovered in mammals more than 30years ago and considered as one of the eIFs recently shown to play a role in the control of protein synthesis [11]. The majority of eIF6 is present in the cytoplasm of eukaryotic cells, with a smaller but indispensable fraction (~30%) located in the nucleus [12,14]. On the one hand eIF6 functions as an anti-association factor and prevents the assembly of the 60S and 40S ribosomal subunits in the cytoplasm [12, 15], but on the other hand eIF6 is a component of the preribosomal particles and plays an important role in 60S ribosome biogenesis in the nucleolus [13, 16]. Thus, subcellular localization could be crucial for the functional regulation of eIF6. Recently, emerging studies have also reported that eIF6 is an important factor in carcinogenesis and tumor progression [17, 18]. Indeed, a study has emphasized that the total depletion of eIF6 could delay tumorigenesis and reduced tumor growth without negative side effects on normal growth in mice, which suggested that eIF6 might be an impactful therapeutic target for cancer treatment [14]. Furthermore, increasing studies confirm dysregulation of eIF6 has been shown in various cancer entities, such as colorectal carcinoma (CRC) [19], malignant pleural mesothelioma (MPM) [20], ovarian adenocarcinoma (OV) [21], breast cancer (MBC) [22] and non-small cell lung cancer (NSCLC) [23]. Mechanistic studies have revealed that phosphorylation may regulate eIF6 activity, and three critical phosphorylation sites have been verified. Nucleus eIF6 is phosphorylated at Ser-175 and Ser-174 in vitro by the nuclear isoforms of casein kinase 1 (CK1), thus advancing the formation of pre-60S ribosomes in the cytoplasm [13]. In addition to the above two sites, the RACK1-PKCII complex also phosphorylates cytoplasmic eIF6 at position Ser-235, which facilitates protein translation and carcinogenesis [12]. The upstream regulatory mechanism of eIF6 has established that the transcription factor complex GAbinding protein (GABP)binding sites are included in the eIF6 promoter region, thereby regulating eIF6 expression [24]. Furthermore, the Notch1/RBPJ signaling pathway stimulates eIF6 promoter activity, enhancing the expression of eIF6 and the invasiveness of ovarian cancer cells [25]. Regarding the for downstream regulation of eIF6, existing studies have demonstrated that eIF6 could indirectly regulate Wnt/-catenin signaling in CRC cells and affect CDC42 signaling in ovarian cancer cells, which promotes migration and invasion [26, 27]. Recent studies also showed that eIF6 could regulate CASP3-related apoptosis signaling in NSCLC and activate multiple AKT-related cancer signaling pathways in CRC [23, 28]. The above evidence suggests that eIF6 might be a potential tumor enhancing factor. However, its definite biological functions and regulatory mechanisms in human carcinoma including HCC remain elusive.

In the present study, we aimed to analyze the potential role and mechanism of eIF6 in the tumorigenesis and progression of HCC. First, we determined that eIF6 was significantly overexpressed in HCC tissues compared with normal tissues by TCGA and GEO databases, which was subsequently verified by IHC and western blot. Based on the analysis of clinicopathological parameters, we also found that high levels of eIF6 had a positive correlation with tumor progression, and eIF6 may serve as a potential diagnostic and prognostic biomarker for HCC patients. In addition, functional studies indicated that eIF6 was a potential driver of tumor biological processes in vitro and in vivo cancer models. Moreover, through signal pathway enrichment analysis and verification based on western blot assay, we confirmed that eIF6 could activate mTOR-related cancer signaling pathways and thereby regulate downstream genes, such as MYC, CDK4, CDK6, CCND1, CASP3 and CTNNBL1, to affect cell proliferation, the cell cycle, apoptosis and invasion in HCC. Our studies elucidated the crucial role of eIF6 in the progression of HCC and provided a potentially valuable biomarker for the diagnosis and prognosis of this cancer.

## Methods

### Data mining and bioinformatics analysis

The gene expression data for hepatocellular cancer (n=374) and adjacent normal tissues (n=50) were downloaded from The Cancer Genome Atlas (TCGA) database (https://www.cancer.gov/tcga). Meanwhile, four eIF6 expression profiles (GSE64041, GSE57957, GSE45436 and GSE14520) were obtained from the Gene Expression Omnibus (GEO) database (http://www.ncbi.nlm.nih.gov/geo). All bioinformatics data were analyzed using R software, and fold change2 and *p*-values0.05 were regarded as statistically significant. The sets of genes which encode proteins interacting with eIF6 were downloaded from UniHI websites (http://www.unihi.org) and then entered into online Metascape (http://www.metascape.org) to perform the pathway enrichment analysis. The data for genes associated with biological function of eIF6 in HCC were downloaded from the R2: Genomics Analysis and Visualization Platform (https://hgserver2.amc.nl/cgi-bin/r2/main.cgi) to analyze the linear correlation of eIF6 with these genes. The KaplanMeier Plotter website (http://kmplot.com/analysis/) was used to analyze overall survival (OS) in patients with HCC. The picture and log rank *p*-values are obtained online.

### Clinical specimens

The clinical specimens, including formalin-fixed paraffin-embedded human HCC specimens (n=68) and fresh surgically removed HCC tissues (n=6), were collected from Affiliated Nanfang Hospital of Southern Medical University (Guangzhou, China) between 2018 and 2020. All tissues were diagnosed as HCC by clinical pathological analysis. Detailed clinical data and information of the specimens were supplied in Additional file 1: Table S1. The study was conducted with written informed consent of all patients and the approval of the Southern Medical University Ethics Committee (Guangzhou, China).

### Cell culture

Human HCC cell lines, SMMC7721, Huh7, HepG2, BEL-7404, BEL-7402, 97H, BEL-7405, HB611 and human normal liver cells (LO2), were obtained from the Culture Collection of the Chinese Academy of Science (Shanghai, China) in 2019. Human HCC cell lines were maintained in RPMI 1640 media (Gibco, USA) containing 10% fetal bovine serum (FBS) (Gibco, USA). LO2 cells were cultured in DMEM (Gibco, USA) supplemented with 10% FBS. All cells were incubated in a 37C thermostatic incubator with 5% CO_2_ (Thermo, USA).

### Western blotting

Total proteins were extracted from tissues or cells using RIPA buffer (KeyGEN, China) containing a protease inhibitor (KeyGEN, China) cocktail. The protein concentration was determined using a BCA Protein Assay kit (KeyGEN, China). Proteins were separated by 1012% SDS-PAGE and transferred onto polyvinylidene difluoride (PVDF) membranes (Millipore, USA). PVDF membranes were blocked with 5% nonfat milk in 1% Tween-PBS (TBST) for 2h at room temperature and then incubated with the specific primary antibodies (Table 1) overnight at 4C. After washing three times for 10min each in TBST, membranes were incubated with their respective second antibodies (Table 1) for 1h at room temperature. Blots were probed using the Pierce ECL Western Blotting Substrate (KeyGEN, China) and chemiluminescence imaging system (Tanon, China). The grey values of the protein bands were quantified by ImageJ software and -actin was used as the internal control.

**Table 1:** The antibodies used in the experiments

Antibody	No	Company	Country
Anti-eIF6	HPA040873	Sigma	USA
Anti--actin	#4970	CST	USA
Anti-mTOR	#2972	CST	USA
Anti-p-mTOR ^Ser2448^	#2971	CST	USA
Anti-Caspase-3	#9662	CST	USA
Anti-Cleaved-Caspase-3	#9664	CST	USA
Anti-CDK4	11,0261-AP	Proteintech	USA
Anti-CDK6	14,0521-AP	Proteintech	USA
Anti-CCND1	26,9391-AP	Proteintech	USA
Anti-MYC	16,2861-AP	Proteintech	USA
Anti-CTNNBL1	13,6651-AP	Proteintech	USA
Anti-Rabbit antibody	SA00001-2	Proteintech	USA
Anti-Ki-67	ZM-0166	ZSGB-BIO	China

CST: Cell Signaling Technology

### Immunohistochemistry (IHC) staining

The immunohistochemistry (IHC) staining procedure was based on previous methods mentioned in the literature [29]. Antibodies specific to eIF6 and Ki67 (Table 1) were applied according to the manufacturers protocol. IHC score (H-score) was used to quantify the expression of eIF6 based on the literature [23].

### Lentiviral vector transduction

Lentiviral vectors repressing eIF6 were constructed by GenePharma Company (Suzhou, China) and were used to infect HCC cells to establish stable cell lines that repressed eIF6. Cells (110^6^/ well) were seeded into six-well plates and transfected with lentiviral after the confluence of cells reached 60%. Then, transfected cells was selected by RPMI 1640 media containing 4g/mL puromycin for 72h and maintained with 1g/mL puromycin. Transduction efficiency was identified for subsequent experiments by western blotting.

### Cell proliferation assay and colony formation assay

Cell proliferation was measured using a Cell Counting Kit-8 (CCK-8) (Dojindo, Japan). Control and transfected HCC cells at equal densities (1000 cells/well) were seeded into 96-well plates and cultured at 37C with 5% CO_2_. Then, 10l CCK-8 reagent was added to each well at 0, 1, 2, 3, 4 and 5days after plating. The plate was incubated for an additional 2h at 37C and the optical density (OD) was measured at 450nm using automatic enzyme labeling (Molecular Devices, Sunnyvale, CA). Each sample was independently repeated 3 times in triplicate. The colony formation assay was performed as previously described in the literature [30].

### Cell cycle and apoptosis analysis

HCC cells transfected with lentiviral repressing eIF6 or the control group were plated into 6-well plates. After the confluence of cells reached 7080%, the cells were digested with EDTA-free trypsin and collected in binding buffer. Cell cycle and apoptosis assays were performed with the Cell Cycle Detection Kit (KeyGEN, China) and Annexin V-APC/7-AAD Apoptosis Detection Kit (KeyGEN, China) according to the manufacturers instruction, respectively. The flow cytometry result was assessed using a BD FACSCanto II flow cytometer (BD Biosciences) with ModFit LT software (Verity Software House, Topsham, ME). Each assay was repeated three times.

### Cell transwell Matrigel invasion assays

A transwell-Matrigel assay was used to verify cell invasion ability. The special chambers with an 8-m pore size (Corning, USA) were plated into 24-well plates and coated with 50 L of Matrigel (dilutions 1:5 in RPMI 1640 media; BD, USA) for 4h. Treated cells (110^5^/well) suspended in 200 L serum-free medium were added to the upper chamber, and 600 L 1640 medium supplemented with 15% FBS was added to the lower chambers. After culturing in a 37C thermostatic incubator for 24h, the cells that invaded into the underside of the transwell chambers were fixed with 4% paraformaldehyde for 30min and stained with 0.1% crystal violet for 20min at room temperature. The number of invasive cells was counted in 5 visual fields under a microscope (Olympus, Japan).

### Subcutaneous xenograft experiment

Female 45weeks old BALB/c-nu (nude) mice were purchased from the Animal Center of Guangdong Province (Guangzhou, China) and were maintained in the SPF Animal Laboratory. HepG2 cells and Huh7 cells (110^7^ in 100 L PBS per mouse) stably transfected with eIF6 silenced lentiviruses (SheIF6) or control lentiviruses (ShNC) were injected into the subcutaneous tissues of nude mice. The tumor size was measured using Vernier calipers, and the tumor volume was calculated by the formula (V=1/2lengthwidthheight) [31]. After 34weeks, nude mice were sacrificed by anesthesia, and tumors were perfectly excised and then weighed and fixed with 10% neutral-buffered formalin overnight. The 4-m sections prepared were stained with hematoxylineosin (HE) and IHC staining using anti-Ki67 according to standard protocols. All animal experiments were performed in compliance with protocols approved by the Animal Care and Use Committee of Southern Medical University.

### Statistical analysis

All statistical analyses were evaluated using SPSS 22.0 software. Quantitative data from three independent experiments are indicated as the meansstandard (meansSD). One-way ANOVA or two-tailed Students t-test were conducted for the comparisons among groups. Chi-square (^2^) tests were used to analyze relationships between eIF6 expression and clinicopathologic characteristics. The KaplanMeier method was performed for survival analysis. Spearmans correlation coefficient was used to examine the linear relationship between eIF6 and target genes in HCC tissues. p<0.05 was regarded as statistically significant.

## Results

### eIF6 is overexpressed in HCC clinical samples and cell lines

**Fig. 1:**
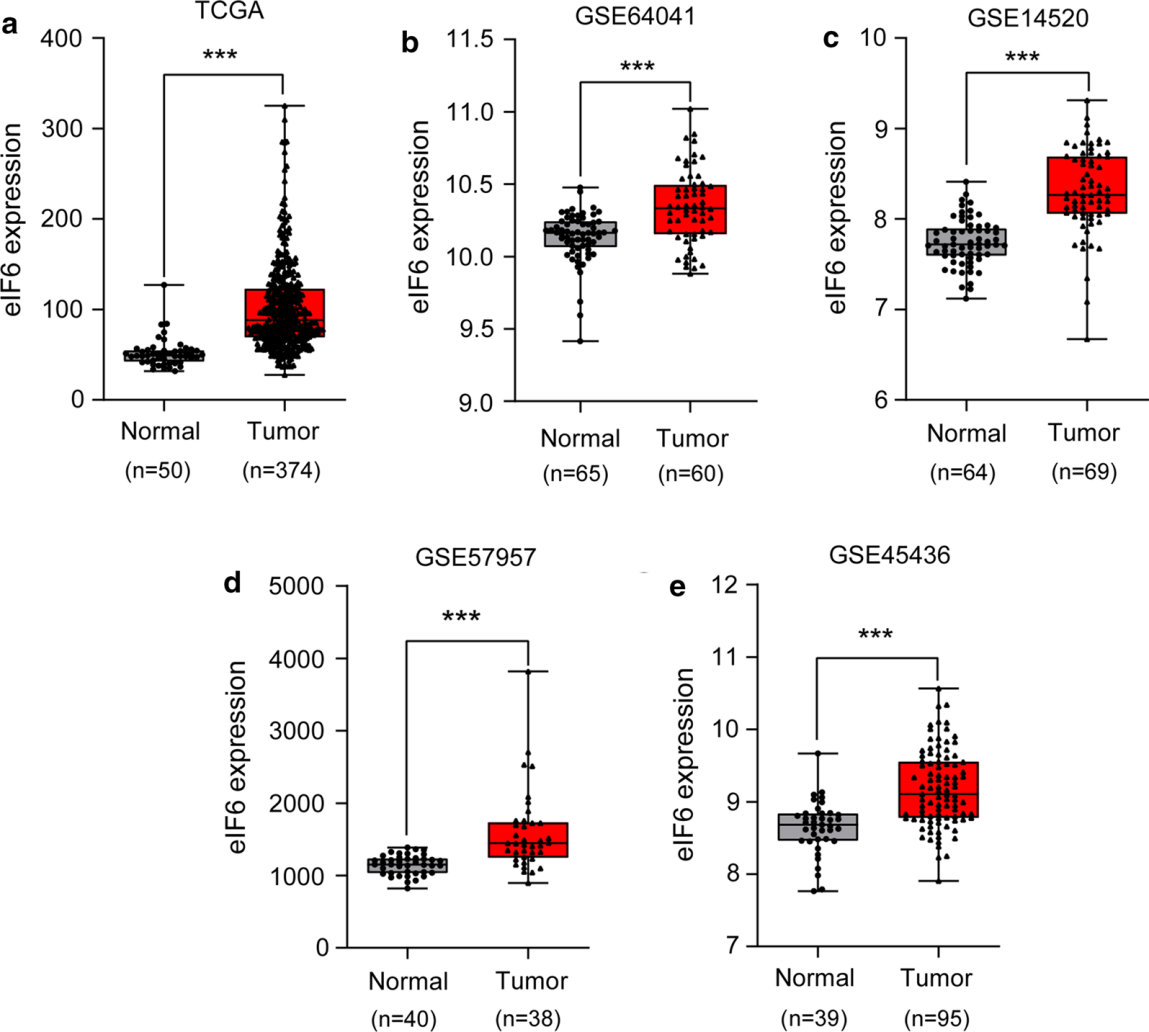
Bioinformatics analysis showed that eIF6 was upregulated in HCC tissues. **a** The expression level of eIF6 in HCC tissues (n=374) was upregulated compared with that of normal tissues (n=50) in the TCGA database. **be** The expression level of eIF6 in HCC tissues was upregulated compared with that of normal tissues in the GEO database, including GSE64041 (Normal=65, Tumor=60), GSE14520 (Normal=64, Tumor=69), GSE57957 (Normal=40, Tumor=38), and GSE45436 (Normal=39, Tumor=95). ***p<0.001. eIF6, eukaryotic translation initiation factor 6; HCC, hepatocellular carcinoma; TCGA, The Cancer Genome Atlas; GEO, Gene Expression Omnibus

We analyzed a total of 424 eIF6 expression profiles from the TCGA liver carcinoma dataset, including 50 normal tissues and 374 tumor tissues. The results showed that eIF6 expression was higher in HCC tissue than in normal liver tissue (p<0.001) (Fig.1a). Furthermore, the expression of eIF6 was also significantly elevated in HCC tissues compared with normal liver tissues by the GEO dataset, including GSE64041, GSE14520, GSE57957, and GSE45436 (all p<0.001) (Fig.1be).

**Fig. 2:**
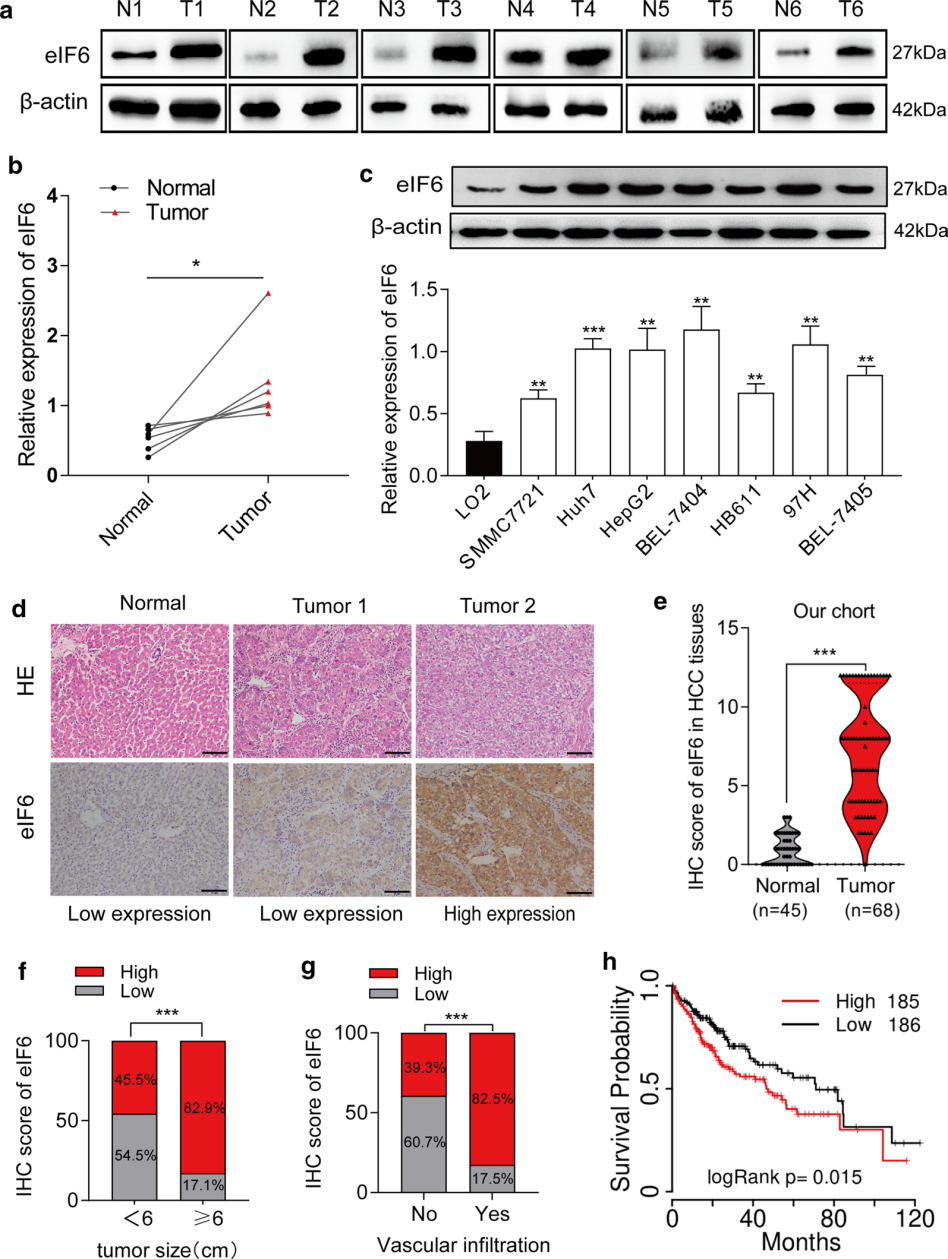
Upregulation of eIF6 is correlated with HCC progression and poor prognosis. **a, b** The expression levels of eIF6 protein were detected by western blot in 6 pairs of fresh surgically resected human HCC tissues. -actin was used as an internal control. The density of eIF6/-actin was calculated as the relative expression levels of eIF6 protein. T, HCC tissues; N, paired adjacent normal liver tissues. **c** Western blot analysis of eIF6 protein levels in 7 HCC cell lines. The data indicate the meansSD (n=3). **d** HE staining and IHC staining of eIF6 antibody in normal liver tissues and HCC tissues. Low expression and high expression images for eIF6 were shown above. Bar=50m. **e** The distribution of scores showed that eIF6 was more highly expressed in HCC (n=68) tissues than in normal liver tissues (n=45). **f** The expression levels of eIF6 had a positive correlation with tumor size. **g** Higher levels of eIF6 in HCC patients were more likely to cause vascular invasion. **h** KaplanMeier analysis of OS based on TCGA database in all 371 patients. Patients with high eIF6 expression (n=185) had a more dismal OS than patients with low eIF6 expression (n=186) in HCC. Color images are available online. The error bar represents the meanSD (n=3). OS, overall survival. *p<0.05; **p<0.01; and ***p<0.001

To further validate the above bioinformatics findings, we evaluated the levels of eIF6 expression in 6 paired fresh HCC tissues and their adjacent normal liver tissues by western blot. As shown in Fig.2a and b, eIF6 levels were evidently increased in fresh HCC tissues (p<0.05). Meanwhile, the relative protein level of eIF6 was significantly upregulated in 7 HCC cell lines compared with that in normal liver cells (LO2) and was higher in Huh7, HepG2 and 97H cells (p<0.01) (Fig.2c). Based on this result, these cell lines with higher level of eIF6 expression were selected for our subsequent experiments. Furthermore, we also performed an IHC assay to verify the above results. The paraffin-embedded tissues were diagnosed as HCC based on HE staining before collection, and eIF6 was mainly located in the cytoplasm, as shown by IHC staining (Fig.2d). Moreover, statistical analysis showed that the expression of eIF6 was distinctly elevated in 64.71% (44/68) of HCC tissues (Table 2), and its high expression was more than that of normal liver tissues (p<0.001) (Fig.2e).

**Table 2:** Clinicopathologic characteristics of eIF6 expression in HCC patients

Clinicopathological variables	N	High expression	Low expression	^2^	p value
**All cases**	**68**	**44**	**24**		
Gender					
Male	59	37 (62.7%)	22 (37.3%)	0.765	0.382
Female	9	7 (77.8%)	2 (22.2%)		
Age					
57	34	21(61.8%)	13(38.2%)	0.258	0.612
>57	34	23(67.6%)	11(32.4%)		
Tumour size (diameter in cm)					
<6	33	15 (45.5%)	18 (54.5%)	10.405	**0.001**
6	35	29 (82.9%)	6 (17.1%)		
Differentiation					
Well	9	6 (66.7%)	3 (33.3%)	0.286	0.355
Moderate	52	35 (67.3%)	17 (32.7%)		
Poor	7	4 (57.1%)	3 (42.9%)		
Vascular invasion					
No	28	11 (39.3%)	17 (60.7%)	13.468	**0.000**
Yes	40	33 (82.5%)	7 (17.5%)		
Lymphatic metastasis					
No	50	31 (62.0%)	19 (38.0%)	0.230	0.632
Yes	18	10 (55.6%)	8 (44.4%)		
Distant metastasis					
No	41	25 (61.0%)	16 (39.0%)	0.629	0.428
Yes	27	19 (70.4%)	8 (29.6%)		
Hepatitis B					
No	19	11 (57.9%)	8 (42.1%)	0.043	0.835
Yes	49	27 (55.1%)	22 (44.9%)		
AFP level ( ng /ml)					
400	53	34 (64.2%)	19 (35.8%)	0.032	0.857
>400	15	10 (66.7%)	5 (33.3%)		
Cirrhosis					
No	33	24 (72.7%)	9 (27.3%)	1.806	0.179
Yes	35	20 (57.1%)	15 (42.9%)		

Bold indicates p-value<0.05

### eIF6 expression is positively correlated with tumor size, vascular invasion and poor prognosis in HCC patients

To further investigate the role of eIF6 in the development of HCC, we analyzed the relationship between the expression of eIF6 and the clinical characteristics of HCC patients from IHC results. The 68 HCC patients were divided into the eIF6-high expression group (n=44) and the eIF6-low expression group (n=24), and the clinical features of these patients are summarized in Table 2. The data show that the expression of eIF6 was significantly correlated with tumor size (p<0.001) (Fig.2f) and vascular invasion (p<0.001) (Fig.2g). In other words, patients with large tumor diameters or vascular invasion occurrences were frequently identified with eIF6 overexpression. However, eIF6 overexpression had no relationship with other clinicopathological features, including gender, age, differentiation, lymphatic metastasis, distant metastasis, HBV infection, AFP level, and liver cirrhosis occurrence. KaplanMeier survival curves indicated that overall survival (OS) time of the eIF6 high expression group was significantly shorter than that of the eIF6-low expression group from the TCGA public database (p=0.015) (Fig.2h). These results indicate that the expression of eIF6 is correlated with tumor size, vascular invasion and poor prognosis in HCC patients.

### eIF6 expression is a reliable clinical diagnostic biomarker for HCC

**Fig. 3:**
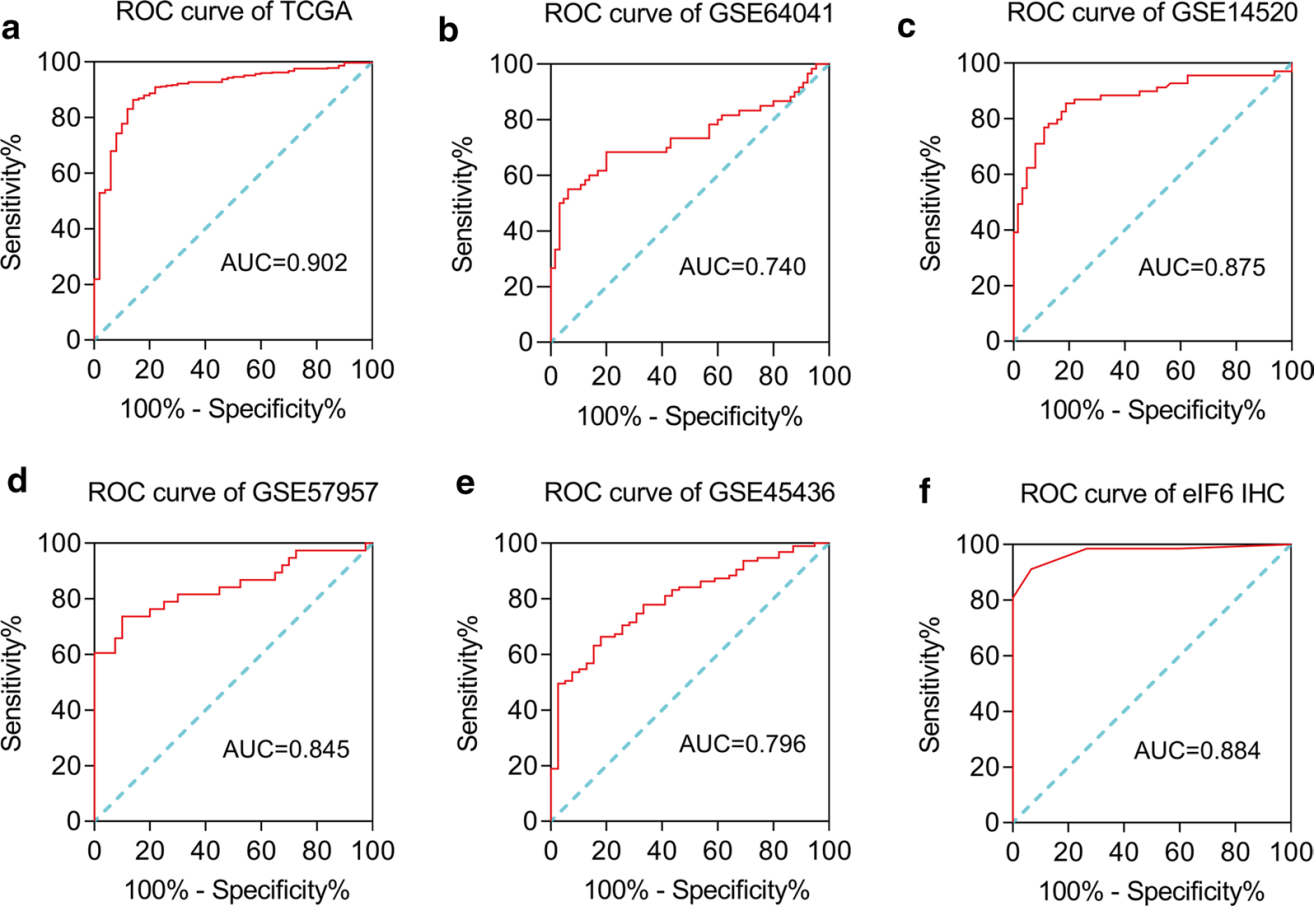
High expression of eIF6 in HCC tissue has reliable diagnostic value. **a** ROC curve for eIF6 expression in normal tissue and HCC tissues from TCGA database, AUC=0.902. **be** ROC curve for eIF6 expression in the following datasets from the GEO database: GSE64041 (AUC=0.740), GSE14520 (AUC=0.875), GSE57957 (AUC=0.845) and GSE45436 (AUC=0.796). f Validation of eIF6 diagnosis value was confirmed in our IHC results of HCC, AUC=0.884. ROC, Receiver Operating Characteristic; AUC, Area Under Curve

We established ROC curves to evaluate the clinical diagnostic value of eIF6 in HCC. The area under the ROC curve (AUC) from TCGA was 0.902 and indicated a high diagnostic power (Fig.3a). Meanwhile, the AUC values from GSE64041, GSE14520, GSE57957 and GSE45436 further validated this result (Fig.3be). The ROC curve of the eIF6 protein also showed authentic diagnostic value in our IHC results (AUC=0.884) (Fig.3f).

### Knockdown of eIF6 inhibits the malignant progression of HCC cells in vitro

**Fig. 4:**
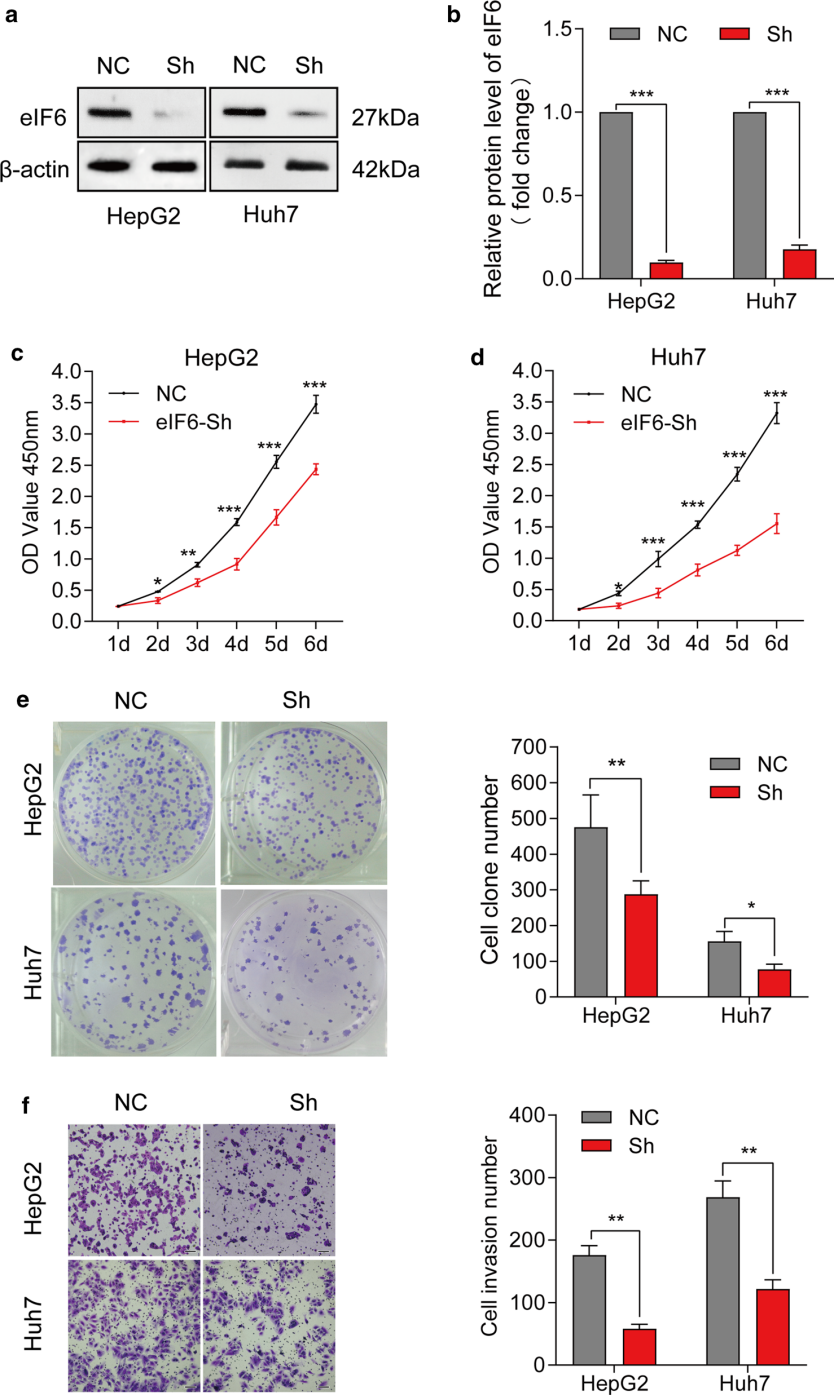
Knockdown of eIF6 inhibited the proliferation and invasion of HCC cells in vitro. **a** Western blot analysis identified the efficiency of knockdown eIF6 lentiviruses in HCC cells. **b** The blot density of the western blotting was quantified using the ImageJ software. -actin was used as the loading control. **c, d** CCK-8 assays were used to determine the cell proliferation capacity in HepG2 and Huh-7 cells infected with knockdown eIF6 lentiviruses. **e** Plate colony formation assays of NC cells and Sh-eIF6 cells (left). The number of colony formation was counted after 15days (right). **f** Transwell-Matrigel invasion assays of NC cells and Sh-eIF6 cells (left). The number of cells invaded after 24h was counted in five randomly selected microscopic fields (right). Bar=100m. The error bar represents the meanSD (n=3). *p<0.05; **p<0.01; and ***p<0.001

To determine the possible biological role of eIF6 in HCC cells, we constructed stable eIF6 knockdown HepG2 and Huh7 cell lines. The infection efficiency was measured by western blotting (p<0.001) (Fig.4a, b). The results of CCK-8 assays revealed that the proliferation activity of eIF6 knockdown cells was deeply decreased compared with that of their control cells (Fig.4c, d). The quantitative analysis from plate colony assays suggested that the number of colonies formed by eIF6 knockdown cells was markedly lower than that of the control cells (p<0.05) (Fig.4e). We then conducted Transwell-Matrigel assays to study effect of eIF6 knockdown on tumor invasion in vitro. The number of cell invasion in the control and Sh-eIF6 groups indicated that eIF6 knockdown dramatically decreased the invasion capacity of HCC cells in vitro (p<0.001) (Fig.4f).

**Fig. 5:**
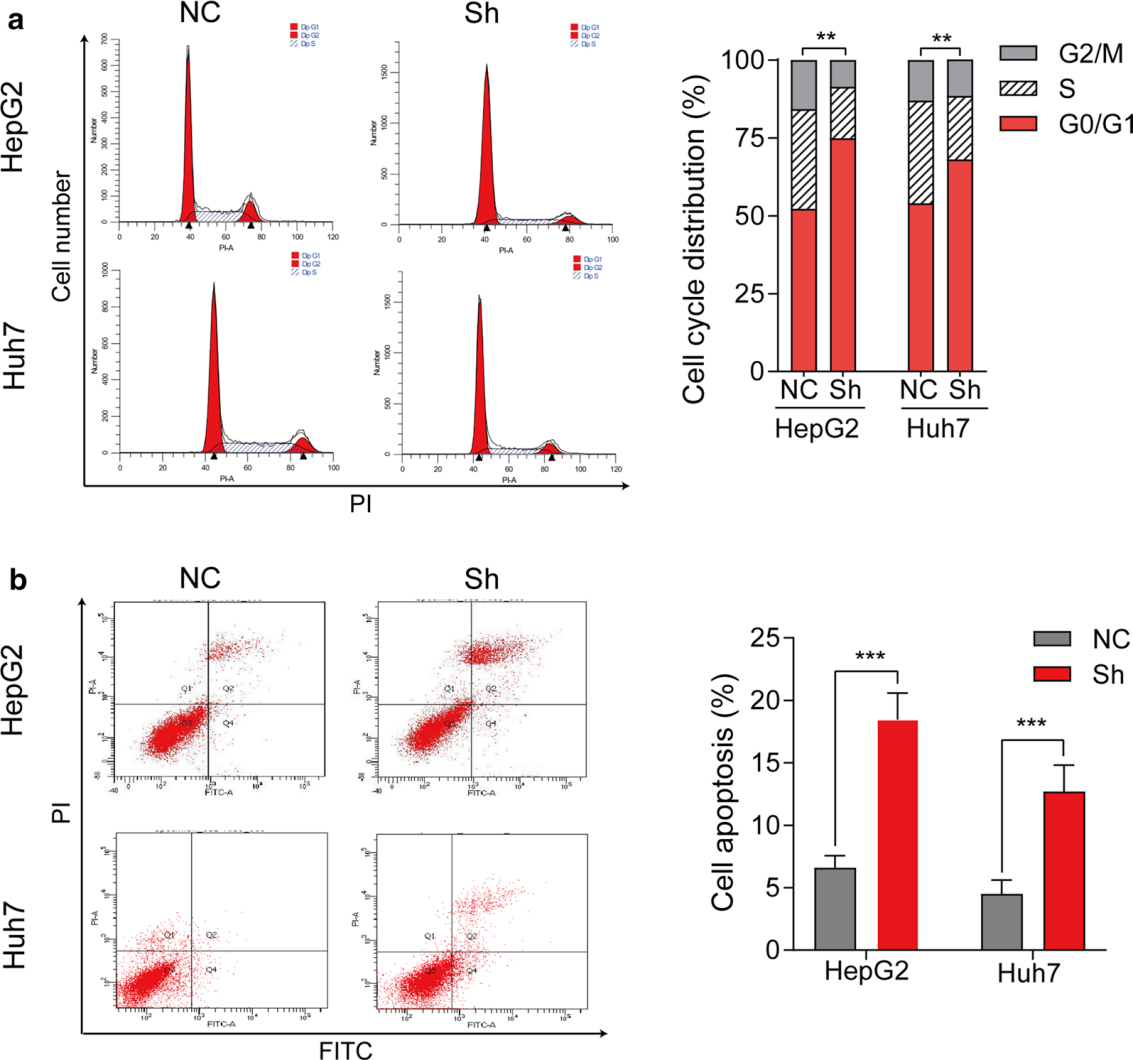
Knockdown of eIF6 induces cell cycle arrest and apoptosis. **a** Cell cycle assay of NC cells and Sh-eIF6 cells was performed by flow cytometry (left). Cell cycle distribution was analyzed from three independent experiments (right). **b** Cell apoptosis assay was performed by flow cytometry (left). Cell apoptosis rate was calculated from three independent experiments (right). The error bar represents the meanSD (n=3). **p<0.01; ***p<0.001

We also detected the effect of eIF6 on apoptosis and the cell cycle in HepG2 and Huh7 cells by flow cytometry assay. As shown in Fig.5a, knockdown of eIF6 increased the proportion of HepG2 and Huh7 cells in G0/G1 phase, but decreased the proportion of cells in S phase, which was similar to the proliferation assay results. For cell apoptosis, knockdown of eIF6 induced a significant increase in the total apoptosis rate in HepG2 cells (p<0.01) and Huh7 cells (p<0. 01) (Fig.5b). Accordingly, these results showed that the increase in cell cycle G0/G1-phase arrest and apoptosis might contribute to the suppression of cell proliferation after depletion of eIF6. Furthermore, similar results were obtained in 97H cells (Additional file 2: Figure S1 and Figure S2). All the above results suggested that eIF6 may promote HCC cells proliferation and invasion in vitro.

### Knockdown of eIF6 arrested tumor growth in a nude subcutaneous xenograft model.

**Fig. 6:**
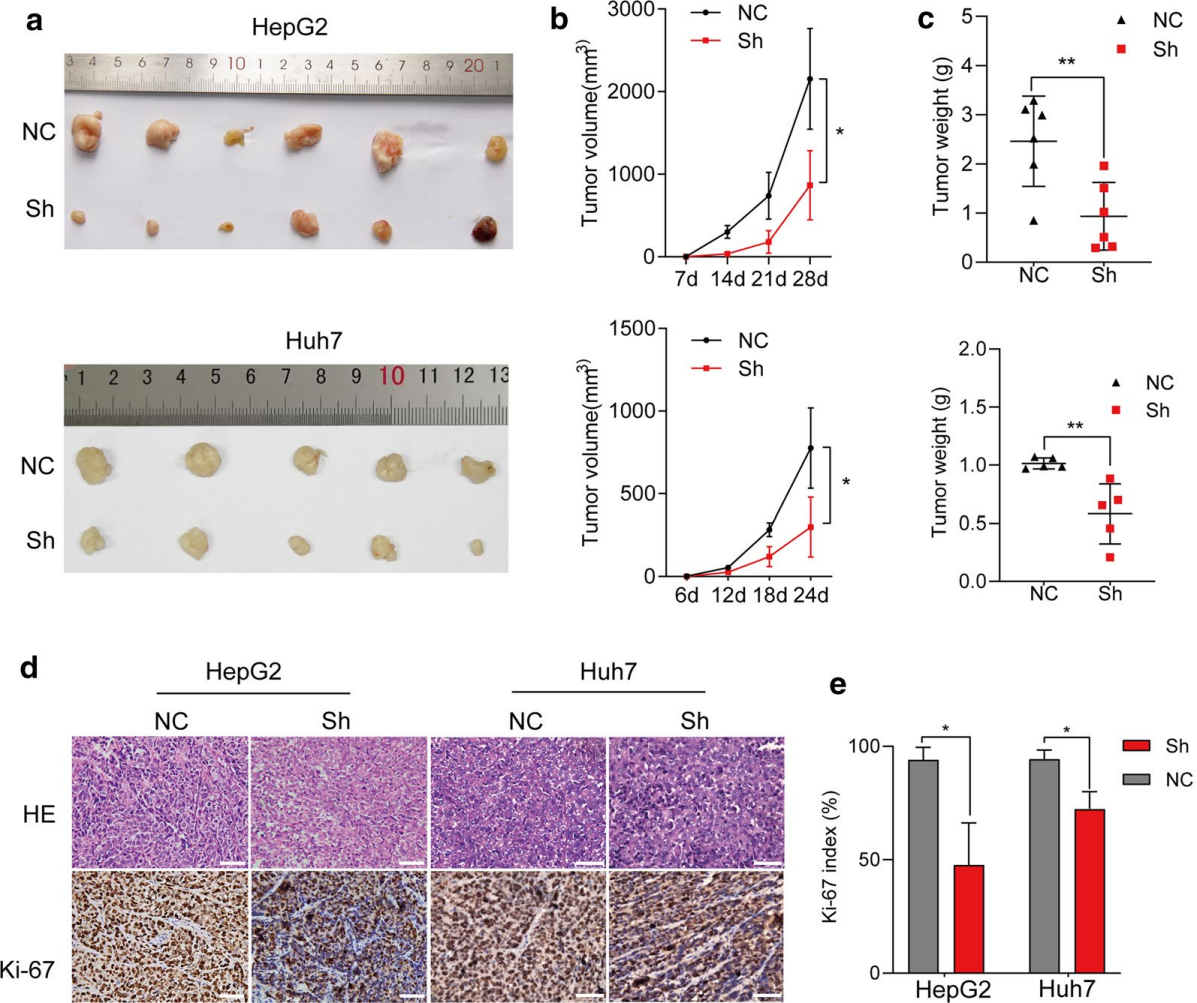
Knockdown of eIF6 in HCC cells reduced the capacity of tumorigenesis in vivo. **a** Images of tumors formed in BALB/c nude mice injected subcutaneously with eIF6-knockdown HepG2 cells (up, n=6 for each group) and Huh7 cells (down, n=5 for each group). **b** The tumor volumes were calculated on the indicated days. **c** The tumor weights were measured after perfect excision from nude mice. **d** HE staining and IHC staining of Ki-67 antibody in sections of subcutaneous tumors. Bar=50m. **e** The Ki-67 index was calculated according to the number of Ki-67-positive cells divided by the number of total cells100%. The error bar represents the meanSD. *p<0.05; **p<0.01

The effect of eIF6 on HCC cell growth was further confirmed in a nude mouse xenograft model. Cells treated with the eIF6 knockdown (Sh) or control (NC) were injected into nude subcutaneous tissue, and the tumor size was measured 23days. Cells in Sh groups injected into nude mice developed smaller (p<0.05) and lighter tumors (p<0.01) with slower growth rate compared with those in the controls (Fig.6ac). Finally, IHC staining of Ki-67 antibody indicated that the Ki-67 index in the Sh groups was lower than those that in the control groups (p<0.05) (Fig.6d, e), further confirming that eIF6 knockdown inhibited HCC cell proliferation in vivo.

### The potential regulatory network and functional enrichment analysis of eIF6 in HCC

**Fig. 7:**
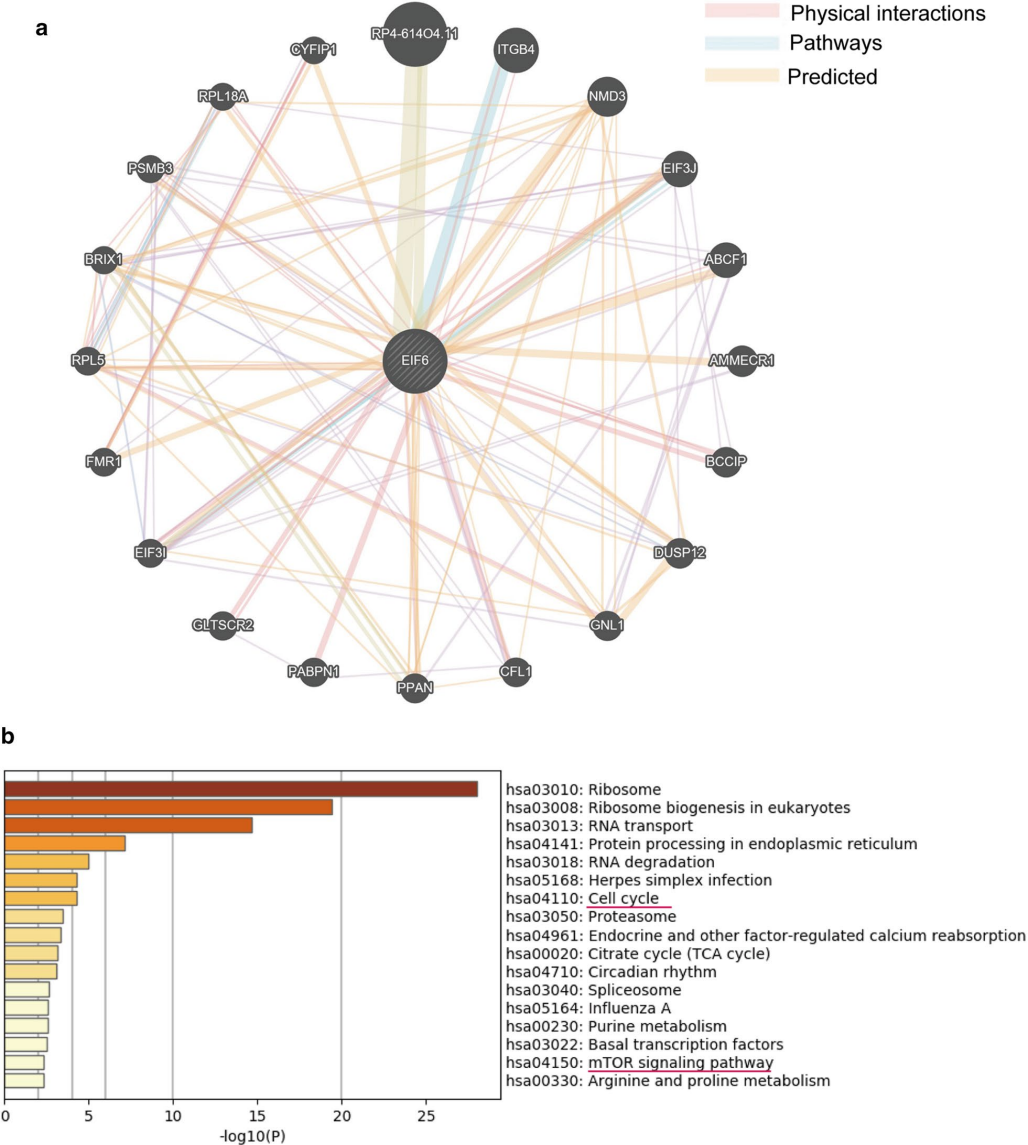
The potential enrichment pathways of elF6 were predicted by the UniHI and Metascape databases. **a** The potential regulation of eIF6 in HCC. eIF6-centered genegene functional interaction network showing the genes with physical interactions, shared signaling pathways, and predicted interactions with eIF6. Color images are available online. **b** The signaling pathway enrichment analysis showed that eIF6 was associated with the cell cycle and mTOR signaling pathways

To further investigate the potential signaling pathways by which eIF6 promoted tumor malignant progression, we examined 355 eIF6-associated proteins using UniHI websites (the details given in Additional file 1: Table S2) and then constructed an eIF6 centered network showing the top 20 proteins using GeneMANIA (Fig.7a). Subsequently, we submitted them to the Metascape online website for pathway enrichment analysis. The results showed that the associated proteins received a high enrichment in the cell cycle (p<0.001) and mTOR signaling pathways (p=0.00338) (Fig.7b, and the details are shown in Additional file 1: Table S3), which are associated with the malignant proliferation of tumor cells.

### eIF6 activated mTOR-related cancer signaling pathways in HCC progression

**Fig. 8:**
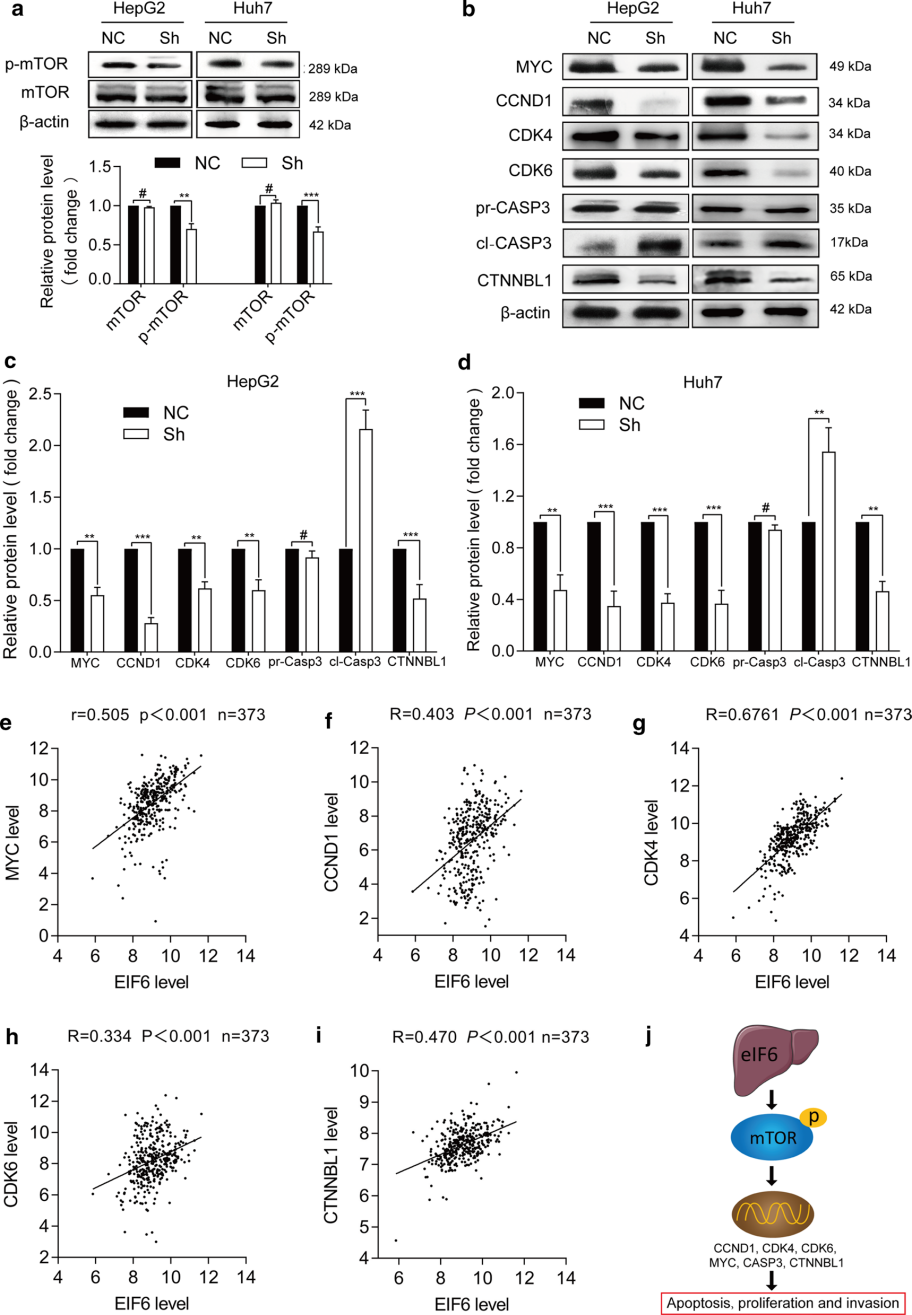
eIF6 activated mTOR-related cancer signaling pathways in HCC progression. **a** The expression level of mTOR-related markers was determined by western blotting (up). The blot density of the western blotting was quantified using the ImageJ software. -actin was used as the loading control (down). **b** Western blotting was used to measure the expression levels of cell cycle, apoptosis and invasion regulators, including MYC, CCND1, CDK4, CDK6, cleaved-CASP3 (cl-CASP3), pro-CASP3 (pr-CASP3) and CTNNBL1. The blot density of the western blotting in HepG2 cells (**c**) and Huh-7 cells (**d**) was quantified using the ImageJ software. **ei** Analysis of the correlation coefficient between eIF6 and cell progression-related genes shown by western blot analysis using the R2 database (n=373). **j** A working model by which eIF6 activated mTOR-related cancer signaling pathways in HCC progression. ^#^p>0.05; *p<0.05; **p<0.01; and ***p<0.001

To confirm above result of enrichment analysis, we examined the effect of eIF6 knockdown on mTOR activity by western blotting. As shown in Fig.8a, eIF6 knockdown decreased the phosphorylated-mTOR (p-mTOR) level in HepG2 and Huh-7 cell lines, which indicating an upstream regulatory role of eIF6 on p-mTOR. This result was further confirmed in 97H cells (Additional file 2: Figure S3). At the same time, cell cycle and invasion regulators, including MYC, CCND1, CDK4, CDK6 and CTNNBL1, were downregulated after eIF6 knockdown, while the apoptosis regulator cleaved-CASP3 was upregulated (Fig.8bd). The detection of a coefficient correlation between eIF6 and the above related markers showed that eIF6 expression was positively correlated with MYC, CCND1, CDK4, CDK6 and CTNNBL1 (Fig.8ei), which was consistent with the western blot results. Therefore, in this part of study, we concluded that eIF6 activated mTOR-related multiple cancer signaling pathways to promote the malignant progression of human HCC (Fig.8j).

### Discussion

Liver cancer is one of the most common malignancies with a high mortality rate. According to the latest cancer statistics of 2020, liver cancer is increasing by 2% to 3% annually with the most rapid speed during 2007 through 2016, and the 5-year relative survival rate is especially dismal [32]. Although diagnostic techniques and therapeutic methods have become more abundant for liver cancer, the improvement of its prognosis remains disappointing [33]. In recent years, exploring specific biomarkers and applying them to clinical prognosis evaluation have become one of the leading research hotspots of cancer [34].

eIFs play important roles in controlling the translation of proteins regulating cell growth, apoptosis, and malignant transformation [35, 36]. As a result of characteristics of active protein synthesis in tumor cells, eIFs are considered to be dysregulated and conducive to malignant progression during carcinogenesis. Indeed, relevant evidences have demonstrated that eIFs overexpression is an important part of the tumorigenesis in numerous patients. For example, eIF4E overexpression is frequently found in patients with colorectal cancer (CRC) or breast cancer (BC) [37, 38]; eIF5A2 overexpression is obviously associated with the advanced stage of ovarian cancer [39]; eIF3D increases cell cycle progression and motility in prostate cancer (PCa) [40]. However, the roles of eIFs in the development and progression of HCC remain limited.

In recent years, the protein eIF6 became included in the family of eukaryotic translation initiation factors controlling growth factor-induced tumors. It is reported that eIF6, as a dual factor, not only facilitates ribosome biogenesis, but also prevents assembly of the 40S and 60S ribosomal subunit [12, 24]. Notably, earlier studies approved that eIF6 downregulation can cause abnormal embryonic development by the G1/S block of the cell cycle in Xenopus [41], and depletion of eIF6 reduced cell proliferation and viability in Saccharomyces cerevisiae [42]. Moreover, knockout of eIF6 in mice avoided MYC-induced lymphomagenesis and prolonged tumor-free survival tumor growth [14]. These above studies point out the important cellular role of eIF6 in cell cycle progression and tumorigenesis. Furthermore, recent studies approved that eIF6 was dysregulated in various cancers, such as malignant pleural mesothelioma (MPM) [20], Gallbladder cancer (GBC) [43], ovarian serous carcinoma (OV) [21], colorectal cancer (CRC) [19] and non-small cell lung cancer (NSCLC) [23]. In CRC, OV and MPM, eIF6 is overexpressed in tumor tissue compared to non-neoplastic tissue, highlighting that eIF6 as a potential new biomarker had a pivotal contribution to pathologic process [19, 21, 23]. In this study, we firstly provided evidence that eIF6 was overexpressed in HCC compared to non-neoplastic liver tissue, and that it might be an important clinical diagnostic biomarker for HCC patients. Our TCGA data set indicated eIF6 overexpression reduced patients overall survival in HCC, which was in accord with existing results from NSCLC and OV [21, 23], highly suggesting that it was a predictor for overall survival in HCC. Thus, examining the RNA or protein quantification of eIF6 could contribute to predict the occurrence of HCC, and directly targeting eIF6 by genetic interference technique might improve therapeutic efficacy.

To further confirm eIF6 as a novel promising target for HCC, we performed knockdown experiments to investigate the biological function of eIF6 in vivo and in vitro cancer model. After successful knockdown of eIF6, the cell growth rate, tumorigenesis and invasive capability of HCC cells were significantly suppressed. This confirms previous reported studies, where eIF6 knockdown in CRC cells significantly reduced proliferation and colonogenicity [19], while eIF6 overexpression enhanced the migratory phenotype by augmenting CDC42 translation in ovarian cancer [26]. The occurrence of tumor is related to not only abnormal proliferation and differentiation of cells but also abnormal apoptosis. Thus, the spontaneous apoptosis of malignant tumor may involve the therapeutic effect of tumor regression [44]. In our study, we also analyzed apoptosis levels, and found that eIF6 knockdown significantly increased apoptosis ratio in transfected HCC cells. According to a published data, eIF6 silencing not only obviously induces CASP3-dependent apoptosis in NSCLC cells but also elevates SA--Gal activity and p21 levels, which is not conducive to the induction of cellular senescence pathways [45], suggesting that eIF6 might have initiated cell death, but this effect are prevented because of effector CASP3 inhibition, thereby cells emit persistent mitogen signaling and develop into an excessive proliferating population. However, specific molecular mechanisms of eIF6 in these biological processes seem to be indecisive and remain to be fully elucidated.

The current study revealed that eIF6-related signaling pathways activated in HCC were enriched in ribosome biogenesis, cell cycle and mTOR-related cancer signaling pathway, which may stimulate tumor proliferation and invasion. The mTOR pathway plays a crucial role in adapting ribosome biogenesis, gene expression and protein translation to surrounding conditions of the cells [43], and the phosphorylation of mTOR signaling pathway molecules and the activation of its downstream proteins promote the proliferation, metabolism and angiogenesis in many cancers [46, 47]. Contrarily, suppression of mTOR effectively inhibits tumor growth and prolongs survival in cancers [48]. Thus, targeting mTOR can contribute to explore novel therapeutic strategies of anticancer. The eIF signaling cascade is mainly regulated via the PI3K/AKT/mTOR pathway due to its vital role in regulating cell growth and proliferation [49, 50]. Moreover, it is confirmed that eIF6 can positively regulate AKT-related cancer signaling and contribute to the malignant behavior of CRC [28]. Most strikingly, an immunohistochemical study in HCC indicated that eIF6 could be involved in the regulation of mTOR pathway [51], but this was not verified by sufficient experimental data. We detected with western blot that phosphorylated mTOR (p-mTOR) was downregulated following attenuation of eIF6 expression, confirming that eIF6 positively regulated mTOR signaling. Consistent with this result, the expression level of eIF6 mRNA had a positive correlation with the mRNAs involved in the arrest of the G0/G1 cell cycle, proliferation, and invasion, including MYC, CDK4, CDK6, CCND1 and CTNNBL1, while a negative correlation with the apoptotic marker cleaved-CASP3, which indicated that some of the biomarkers had altered steady state mRNA levels upon eIF6 overexpression, thereby resulting in upregulation of protein expression. In other words, eIF6 is likely to control their expression via the translational regulation of some transcription factors. Thus, our findings indicated that eIF6 promoted the malignant progression of HCC via the mTOR signaling pathway, which provided a theoretical basis for HCC treatment by targeting eIF6.

Although relevant studies have confirmed that the GABP complex regulates the transcription of eIF6 and that the Notch-l signaling pathway stimulates the activity of eIF6 promoter in lymphoblastoid and ovarian cancer cell lines [24, 25]. However, the upstream regulatory mechanism involved in eIF6 has not been investigated in our study and further study on this mechanism is required to be performed. Moreover, we should treat HCC cells or animal models with mTOR pathway inhibitors or stimulators and further detect the precise and detailed regulatory mechanisms between eIF6 and mTOR-related signal molecules.

## Conclusion

Our findings illustrated that eIF6 overexpression was not only observed in HCC, but also correlated with malignant progression and poorer prognosis. eIF6 might serve as a promising biomarker for the HCC diagnosis in immunohistochemistry and its regulation could be used in the future as a novel potential therapeutic target in HCC.

## Supplementary Information

**Additional file 1: Table S1.** Detailed clinical data and information of the specimens. **Table S2.** eIF6 interacting proteins analysis using UniHI websites. **Table S3.** Signaling pathway enrichment analysis using Metascape online website.

**Additional file 2: Figure S1.** Knockdown of eIF6 inhibited the proliferation and invasion of 97H cells in vitro. **Figure S2.** Knockdown of eIF6 induces cell cycle arrest and apoptosis of 97H cells. **Figure S3.** eIF6 activated mTOR-related cancer signaling pathways in 97H cells.

## Data Availability

All data generated or analysed during this study are included in this published article.

## Abbreviations

eIF6: Eukaryotic translation initiation factor 6
HCC: Hepatocellular carcinoma
TCGA: The Cancer Genome Atlas
GEO: Gene Expression Omnibus
ROC: Receiver operating characteristic curve
CCND1: Cyclin D1
CDK4: Cyclin dependent kinase 4
CDK6: Cyclin dependent kinase 6
MYC: MYC proto-oncogene
CTNNBL1: Catenin beta like 1
IHC: Immunohistochemistry
FBS: Fetal bovine serum
MF: Molecular function
BP: Biological process
CC: Cellular component

## References

[1] Bray F, Ferlay J, Soerjomataram I, Siegel RL, Torre LA, Jemal A (2018). Global cancer statistics 2018: GLOBOCAN estimates of incidence and mortality worldwide for 36 cancers in 185 countries. CA Cancer J Clin.

[2] Islami F, Goding SA, Miller KD, Siegel RL, Fedewa SA, Jacobs EJ (2018). Proportion and number of cancer cases and deaths attributable to potentially modifiable risk factors in the United States. CA Cancer J Clin.

[3] Allemani C, Matsuda T, Di Carlo V, Harewood R, Matz M, Niksic M (2018). Global surveillance of trends in cancer survival 200014 (CONCORD-3): analysis of individual records for 37 513 025 patients diagnosed with one of 18 cancers from 322 population-based registries in 71 countries. Lancet.

[4] Ramamurthy VP, Ramalingam S, Kwegyir-Afful AK, Hussain A, Njar VC (2017). Targeting of protein translation as a new treatment paradigm for prostate cancer. Curr Opin Oncol.

[5] Showkat M, Beigh MA, Andrabi KI (2014). mTOR Signaling in Protein Translation Regulation: Implications in Cancer Genesis and Therapeutic Interventions. Mol Biol Int..

[6] Graff JR, Konicek BW, Lynch RL, Dumstorf CA, Dowless MS, Mcnulty AM (2009). eIF4E activation is commonly elevated in advanced human prostate cancers and significantly related to reduced patient survival. Cancer Res.

[7] Smolle MA, Czapiewski P, Lapinska-Szumczyk S, Majewska H, Supernat A, Zaczek A (2019). The Prognostic Significance of Eukaryotic Translation Initiation Factors (eIFs) in Endometrial Cancer. Int J Mol Sci.

[8] Bitterman PB, Polunovsky VA (2015). eIF4E-mediated translational control of cancer incidence. Biochim Biophys Acta.

[9] Niu Z, Xu P, Zhu D, Tang W, Ji M, Lin Q (2018). Integrin beta1 mediates 5-fluorouracil chemoresistance under translational control of eIF4E in colorectal cancer. Int J Clin Exp Pathol.

[10] Golob-Schwarzl N, Puchas P, Gogg-Kamerer M, Weichert W, Goppert B, Haybaeck J (2020). New Pancreatic Cancer Biomarkers eIF1, eIF2D, eIF3C and eIF6 Play a Major Role in Translational Control in Ductal Adenocarcinoma. Anticancer Res.

[11] Biffo S, Sanvito F, Costa S, Preve L, Pignatelli R, Spinardi L (1997). Isolation of a novel beta4 integrin-binding protein (p27(BBP)) highly expressed in epithelial cells. J Biol Chem.

[12] Ceci M, Gaviraghi C, Gorrini C, Sala LA, Offenhauser N, Marchisio PC (2003). Release of eIF6 (p27BBP) from the 60S subunit allows 80S ribosome assembly. Nature.

[13] Miluzio A, Beugnet A, Volta V, Biffo S (2009). Eukaryotic initiation factor 6 mediates a continuum between 60S ribosome biogenesis and translation. EMBO Rep.

[14] Miluzio A, Beugnet A, Grosso S, Brina D, Mancino M, Campaner S (2011). Impairment of cytoplasmic eIF6 activity restricts lymphomagenesis and tumor progression without affecting normal growth. Cancer Cell.

[15] Valenzuela DM, Chaudhuri A, Maitra U (1982). Eukaryotic ribosomal subunit anti-association activity of calf liver is contained in a single polypeptide chain protein of Mr = 25,500 (eukaryotic initiation factor 6). J Biol Chem.

[16] Sanvito F, Piatti S, Villa A, Bossi M, Lucchini G, Marchisio PC (1999). The beta4 integrin interactor p27(BBP/eIF6) is an essential nuclear matrix protein involved in 60S ribosomal subunit assembly. J Cell Biol.

[17] Brina D, Miluzio A, Ricciardi S, Biffo S (2015). eIF6 anti-association activity is required for ribosome biogenesis, translational control and tumor progression. Biochim Biophys Acta.

[18] Brina D, Grosso S, Miluzio A, Biffo S (2011). Translational control by 80S formation and 60S availability: the central role of eIF6, a rate limiting factor in cell cycle progression and tumorigenesis. Cell Cycle.

[19] Golob-Schwarzl N, Schweiger C, Koller C, Krassnig S, Gogg-Kamerer M, Gantenbein N (2017). Separation of low and high grade colon and rectum carcinoma by eukaryotic translation initiation factors 1, 5 and 6. Oncotarget.

[20] Miluzio A, Oliveto S, Pesce E, Mutti L, Murer B, Grosso S (2015). Expression and activity of eIF6 trigger malignant pleural mesothelioma growth in vivo. Oncotarget.

[21] Flavin RJ, Smyth PC, Finn SP, Laios A, O'Toole SA, Barrett C (2008). Altered eIF6 and Dicer expression is associated with clinicopathological features in ovarian serous carcinoma patients. Mod Pathol.

[22] Gatza ML, Silva GO, Parker JS, Fan C, Perou CM (2014). An integrated genomics approach identifies drivers of proliferation in luminal-subtype human breast cancer. Nat Genet.

[23] Gantenbein N, Bernhart E, Anders I, Golob-Schwarzl N, Krassnig S, Wodlej C (2018). Influence of eukaryotic translation initiation factor 6 on non-small cell lung cancer development and progression. Eur J Cancer.

[24] Donadini A, Giacopelli F, Ravazzolo R, Gandin V, Marchisio PC, Biffo S (2006). GABP complex regulates transcription of eIF6 (p27BBP), an essential trans-acting factor in ribosome biogenesis. FEBS Lett.

[25] Benelli D, Cialfi S, Pinzaglia M, Talora C, Londei P (2012). The translation factor eIF6 is a Notch-dependent regulator of cell migration and invasion. PLoS ONE..

[26] Pinzaglia M, Montaldo C, Polinari D, Simone M, La Teana A, Tripodi M (2015). EIF6 over-expression increases the motility and invasiveness of cancer cells by modulating the expression of a critical subset of membrane-bound proteins. BMC Cancer.

[27] Ji Y, Shah S, Soanes K, Islam MN, Hoxter B, Biffo S (2008). Eukaryotic initiation factor 6 selectively regulates Wnt signaling and beta-catenin protein synthesis. Oncogene.

[28] Lin J, Yu X, Xie L, Wang P, Li T, Xiao Y (2019). eIF6 Promotes Colorectal Cancer Proliferation and Invasion by Regulating AKT-Related Signaling Pathways. J Biomed Nanotechnol.

[29] Xiang J, Fang L, Luo Y, Yang Z, Liao Y, Cui J (2014). Levels of human replication factor C4, a clamp loader, correlate with tumor progression and predict the prognosis for colorectal cancer. J Transl Med.

[30] Li J, Xia R, Liu T, Cai X, Geng G (2020). LncRNA-ATB promotes lung squamous carcinoma cell proliferation, migration, and invasion by targeting microRNA-590-5p/NF90 Axis. DNA Cell Biol.

[31] Lin C, Zhang J, Lu Y, Li X, Zhang W, Zhang W (2018). NIT1 suppresses tumour proliferation by activating the TGFbeta1-Smad2/3 signalling pathway in colorectal cancer. Cell Death Dis.

[32] Siegel RL, Miller KD, Jemal A (2020). Cancer statistics, 2020. CA Cancer J Clin.

[33] Pascual S, Herrera I, Irurzun J (2016). New advances in hepatocellular carcinoma. World J Hepatol.

[34] De Stefano F, Chacon E, Turcios L, Marti F, Gedaly R (2018). Novel biomarkers in hepatocellular carcinoma. Dig Liver Dis.

[35] Robichaud N, Sonenberg N, Ruggero D, Schneider RJ (2019). Translational Control in Cancer. Cold Spring Harb Perspect Biol..

[36] Sonenberg N, Hinnebusch AG (2009). Regulation of translation initiation in eukaryotes: mechanisms and biological targets. Cell.

[37] Xu T, Zong Y, Peng L, Kong S, Zhou M, Zou J (2016). Overexpression of eIF4E in colorectal cancer patients is associated with liver metastasis. Onco Targets Ther.

[38] Humphries MP, Sundara RS, Droop A, Suleman C, Carbone C, Nilsson C (2017). A Case-Matched Gender Comparison Transcriptomic Screen Identifies eIF4E and eIF5 as Potential Prognostic Markers in Male Breast Cancer. Clin Cancer Res.

[39] Guan XY, Fung JM, Ma NF, Lau SH, Tai LS, Xie D (2004). Oncogenic role of eIF-5A2 in the development of ovarian cancer. Cancer Res.

[40] Gao Y, Teng J, Hong Y, Qu F, Ren J, Li L (2015). The oncogenic role of EIF3D is associated with increased cell cycle progression and motility in prostate cancer. Med Oncol.

[41] De Marco N, Tussellino M, Vitale A, Campanella C (2011). Eukaryotic initiation factor 6 (eif6) overexpression affects eye development in Xenopus laevis. Differentiation.

[42] Basu U, Si K, Warner JR, Maitra U (2001). The Saccharomyces cerevisiae TIF6 gene encoding translation initiation factor 6 is required for 60S ribosomal subunit biogenesis. Mol Cell Biol.

[43] Golob-Schwarzl N, Wodlej C, Kleinegger F, Gogg-Kamerer M, Birkl-Toeglhofer AM, Petzold J (2019). Eukaryotic translation initiation factor 6 overexpression plays a major role in the translational control of gallbladder cancer. J Cancer Res Clin Oncol.

[44] Evan GI, Vousden KH (2001). Proliferation, cell cycle and apoptosis in cancer. Nature.

[45] Bernhart E, Damm S, Heffeter P, Wintersperger A, Asslaber M, Frank S (2014). Silencing of protein kinase D2 induces glioma cell senescence via p53-dependent and -independent pathways. Neuro Oncol.

[46] Wu ZH, Lin C, Liu CC, Jiang WW, Huang MZ, Liu X (2018). MiR-616-3p promotes angiogenesis and EMT in gastric cancer via the PTEN/AKT/mTOR pathway. Biochem Biophys Res Commun.

[47] Mai S, Xiao R, Shi L, Zhou X, Yang T, Zhang M (2019). MicroRNA-18a promotes cancer progression through SMG1 suppression and mTOR pathway activation in nasopharyngeal carcinoma. Cell Death Dis.

[48] Wang H, Liu Y, Ding J, Huang Y, Liu J, Liu N (2020). Targeting mTOR suppressed colon cancer growth through 4EBP1/eIF4E/PUMA pathway. Cancer Gene Ther.

[49] Zakaria C, Sean P, Hoang HD, Leroux LP, Watson M, Workenhe ST (2018). Active-site mTOR inhibitors augment HSV1-dICP0 infection in cancer cells via dysregulated eIF4E/4E-BP axis. PLoS Pathog..

[50] Harris TE, Chi A, Shabanowitz J, Hunt DF, Rhoads RE, Lawrence JJ (2006). mTOR-dependent stimulation of the association of eIF4G and eIF3 by insulin. EMBO J.

[51] Golob-Schwarzl N, Krassnig S, Toeglhofer AM, Park YN, Gogg-Kamerer M, Vierlinger K (2017). New liver cancer biomarkers: PI3K/AKT/mTOR pathway members and eukaryotic translation initiation factors. Eur J Cancer.

