# Supplementary figures and images for: eIF6 promotes the malignant progression of human hepatocellular carcinoma via the mTOR signaling pathway
Source: J Transl Med. 2021 May 20;19:216. doi: 10.1186/s12967-021-02877-4 (PMC8139032; doi:10.1186/s12967-021-02877-4)

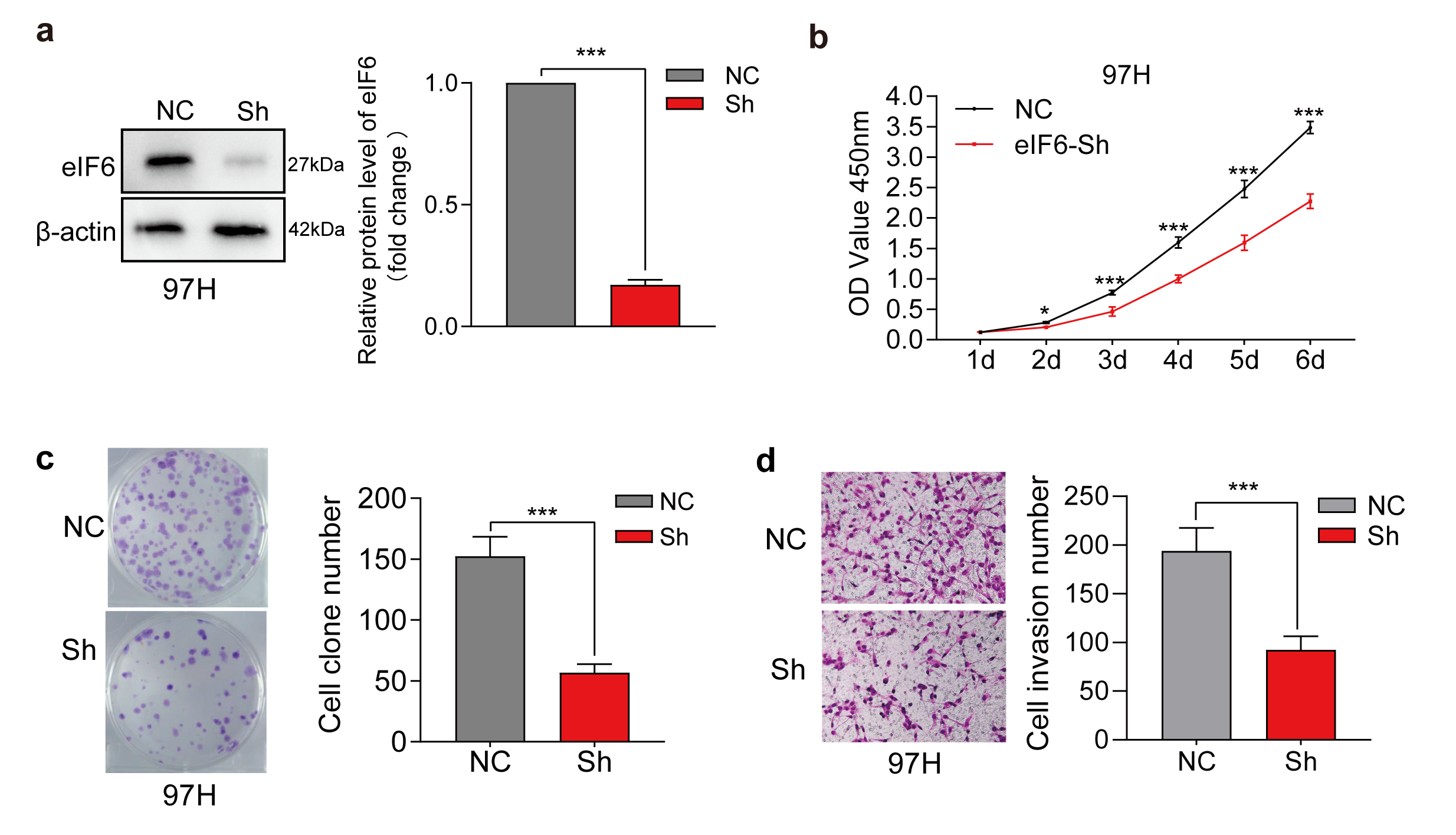

Supplement: Supplementary file 2 — Additional file 2: Figure S1. Knockdown of eIF6 inhibited the proliferation and invasion of 97H cells in vitro. Figure S2. Knockdown of eIF6 induces cell cycle arrest and apoptosis of 97H cells. Figure S3. eIF6 activated mTOR-related cancer signaling pathways in 97H cells. [file 12967_2021_2877_MOESM2_ESM.zip › SunLiping.Figure S1.tif]

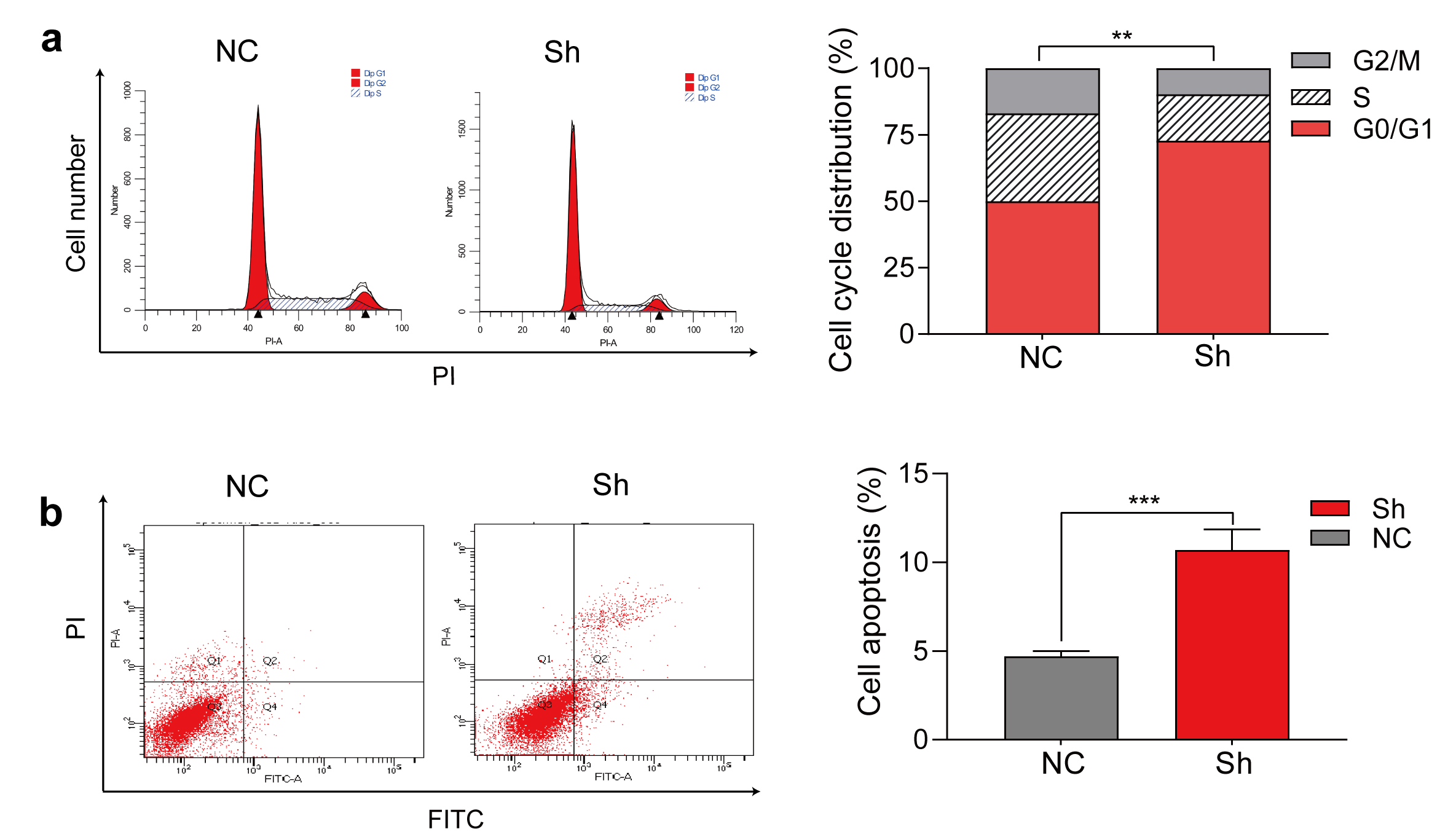

Supplement: Supplementary file 2 — Additional file 2: Figure S1. Knockdown of eIF6 inhibited the proliferation and invasion of 97H cells in vitro. Figure S2. Knockdown of eIF6 induces cell cycle arrest and apoptosis of 97H cells. Figure S3. eIF6 activated mTOR-related cancer signaling pathways in 97H cells. [file 12967_2021_2877_MOESM2_ESM.zip › SunLiping.Figure S2.tif]

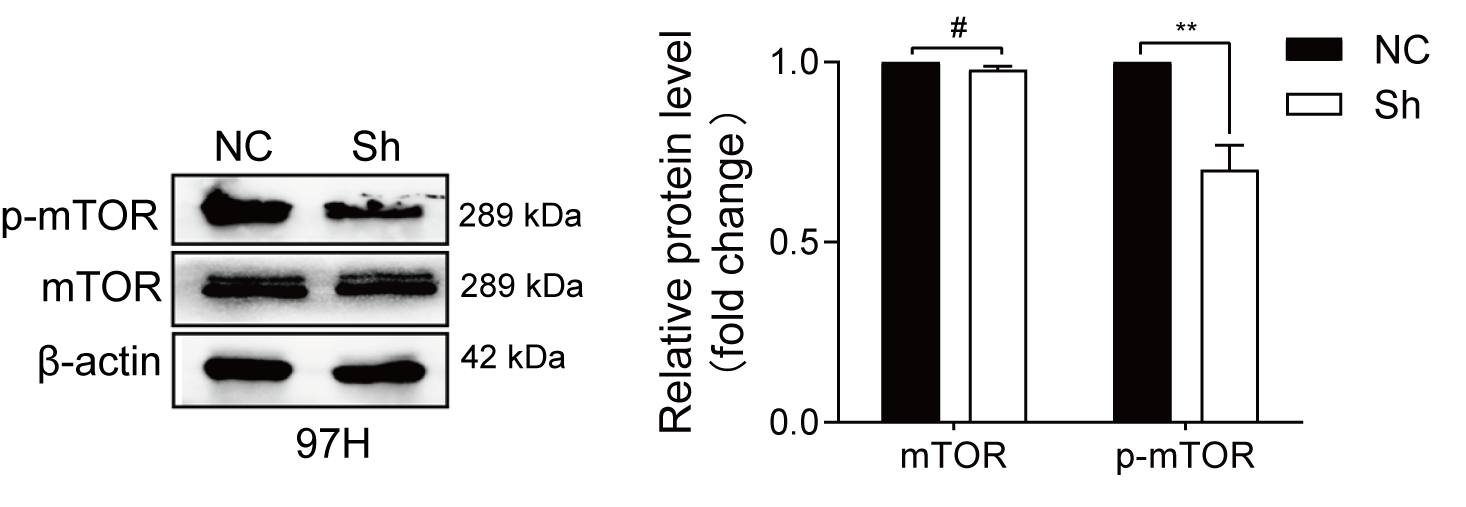

Supplement: Supplementary file 2 — Additional file 2: Figure S1. Knockdown of eIF6 inhibited the proliferation and invasion of 97H cells in vitro. Figure S2. Knockdown of eIF6 induces cell cycle arrest and apoptosis of 97H cells. Figure S3. eIF6 activated mTOR-related cancer signaling pathways in 97H cells. [file 12967_2021_2877_MOESM2_ESM.zip › SunLiping.Figure S3.tif]
